# COVID-19 pandemic impact on psychotropic prescribing for adults with intellectual disability: an observational study in English specialist community services

**DOI:** 10.1192/bjo.2021.1064

**Published:** 2021-12-06

**Authors:** Danial Naqvi, Bhathika Perera, Sarah Mitchell, Rory Sheehan, Rohit Shankar

**Affiliations:** Barnet Enfield and Haringey Mental Health Trust, UK; Cornwall Partnership NHS Foundation Trust, UK; Institute of Psychiatry, Psychology and Neuroscience, King's College, UK; Cornwall Partnership NHS Foundation Trust, UK; and Cornwall Intellectual Disability Equitable Research (CIDER) University of Plymouth Peninsula School of Medicine, UK

**Keywords:** Prescribing, psychotropic medications, intellectual disability, COVID-19 pandemic, autism

## Abstract

**Background:**

Coronavirus disease 2019 (COVID-19) has had a disproportionate impact on people with intellectual disability (PwID). PwID are at higher risk of mental illness and receive psychotropic prescribing ‘off licence' also, to manage distress behaviour. The lockdown and reduction of multidisciplinary face-to-face appointments had an impact on care delivery, the recourse possibly being psychotropic prescribing. It is imperative to comprehend the influence the pandemic had on psychotropic prescribing patterns to enable future planning.

**Aims:**

The aim was to understand the impact of the pandemic by comparing psychotropic prescribing patterns during the England lockdown with the prescribing patterns before lockdown in specialist urban and rural psychiatric services for PwID.

**Method:**

Data was collected from Cornwall (rural) and London (urban) intellectual disability services in England as a service evaluation project to rationalise psychotropic prescribing. PwID in both services open across January 2020 to January 2021 were included. Baseline patient demographics including age, gender, ethnicity, intellectual disability level and neurodevelopmental and psychological comorbidities were collected. Baseline psychotropic prescribing and subsequent % change for each psychotropic group for the two services was compared using Pearson's chi-square and *z-*statistic (two tailed) with significance taken at *P* < 0.05.

**Results:**

The two centres London (*n* = 113) and Cornwall (*n* = 97) were largely comparable but for baseline differences in terms of presence of severe mental illness (37 *v*. 86, *P* < 0.001), challenging behaviour (44 *v*. 57, *P* < 0.05) and attention-deficit hyperactivity disorder (37 *v*. 3, *P* < 0.001). There was an overall increase in psychotropic prescribing during lockdown in urban as compared with rural settings (11% *v*. 2%).

**Conclusions:**

The pandemic caused an increase in psychotropic prescribing associated with lockdown severity and urban settings. Team structures could have played a role.

## Background

The coronavirus disease 2019 (COVID-19) pandemic has had a devastating impact on health outcomes globally. There has been an increasing focus on mental health difficulties. Increasing rates of mental disorders directly or indirectly resulting from the pandemic are being reported.^1,2^ Those with existing mental health problems have been disproportionately affected during the pandemic, with evidence showing the effects of lockdown and restriction exacerbating existing mood disorders and mental illnesses.^3,4^

The pandemic has had an excessive impact on people with intellectual disability (PwID, intellectual disability is also known as learning disability in UK health services). As the pandemic has continued, increased risk of hospital admissions and mortality from COVID-19 among PwID has become clear.^5^ Current evidence shows that there is an overrepresentation of mental health issues and psychotropic prescribing in those PwID succumbing to COVID.^6^ The disproportionate impact of the pandemic on the mental health of PwID and their carers has also been raised.^7,8^

PwID generally have three times higher rates of mental disorders than the general population.^9,10^ The impact of restrictions on day-to-day activities and change to their routine can be an added challenge for PwID affecting their mental well-being. Higher levels of comorbid neurodevelopmental disorders such as autism spectrum disorder (ASD) and attention-deficit hyperactivity disorder (ADHD) and epilepsy can further exacerbate the distress experienced by the person with intellectual disability and their carers.^11^

Before the pandemic, up to one in five PwID were also recognised to have challenging behaviour.^12^ A range of biological, psychological, social and environmental factors play a significant role in precipitating and perpetuating the mental and behavioural disorders. Communication and cognitive deficits, access to fewer coping resources, higher incidence of negative life events, early childhood trauma, impact of attitudes of those surrounding them, and genetic vulnerability to mental health problems are some of the factors that can predispose to mental and behavioural disorders in PwID.^13,14^ Studies have shown that disproportionate numbers of PwID are prescribed psychotropic medications compared with their peers without intellectual disability, particularly without a licensed indication.^15,16^ A major concern over the past decade has been the role of prescribing psychotropic medication in PwID to manage challenging behaviour.^16^ The impact of the pandemic on prescribing patterns for challenging behaviour and mental health issues in PwID has not yet been studied.

## Aims

Comparing pre-COVID psychotropic prescribing patterns with a year after the start of COVID pandemic can show the trajectory of psychotropic prescribing during the pandemic. This will help to explore the impact of the pandemic on mental health and challenging behaviour of PwID even though many other factors can also affect the prescribing pattern such as lack of access to alternative treatment options. Comparing prescribing patterns in two areas affected differently may help to further explore factors that influence prescribing and inform clinical practice as to how best to support those with intellectual disability in the future. The aim of this study was therefore to explore psychotropic prescribing patterns during the pandemic in adults with intellectual disability to understand the impact of COVID and other associated factors affecting prescribing patterns.

## Method

The STROBE checklist was used to guide this retrospective cohort study.^17^

### Study setting

Mental health services for adults with intellectual disability in England are provided by multidisciplinary community teams specialised in diagnosing and treating mental disorders in adults with intellectual disability. Two such services, one urban (London) and one rural (Cornwall), were selected. The London centre has a population of 300 000 with 400 PwID registered with local services. Cornwall is a county in the south west of England with a population of 540 000 with approximately 800 PwID open to the team. Similarities and differences of psychiatric provision in the two settings are provided in Appendix 1. There was a marked reduction in face-to-face contacts and an increase in virtual and telephone consultations during the pandemic for both teams. There was a higher infection rate in London compared with Cornwall during the pandemic resulting in stricter local restrictions in London compared with Cornwall.^18^ Specific details of the UK lockdowns are provided on the Institute For Government website.^19^

### Ethics

The project used anonymised pooled data from the two centres. No individual patient data from each centre was shared with the other. Data was collected as part of ongoing service evaluation and registered as such for both organisations. We also used the NHS Health research authority tool (http://www.hra-decisiontools.org.uk/research/index.html) that confirmed no formal NHS ethics approval was required (Supplementary File 1, available at https://doi.org/10.1192/bjo.2021.1064). No author had access to any patient identifiable information other than to their own clients within their service. To help outline confounders and potential bias, similarities and differences in the service delivery of the two centres were also compared.

Data were collected at two different points. The first data collection point was in January 2020 followed by the second data point in January 2021 as national lockdown restrictions in England were implemented in March 2020. All diagnosis are ICD/DSM validated.^20,21^

The following inclusion criteria was used:

- patient has been with the service since December 2019;
- patient has an intellectual disability;
- patient is on psychotropic medications in January 2020.

The following exclusion criteria was used:

- patient is under the age of 18 years;
- patient has had <2 consultations since December 2019;
- patient died prior to January 2021.

The following information was collated cross sectionally for two data points (January 2020 and January 2021):

- age and gender;
- level of intellectual disability – divided into mild/moderate-severe as per the ICD-10;
- number of patients with ASD and/or ADHD;
- number of patients with severe mental illness – classified as bipolar affective disorder, severe depression, psychosis;
- number of patients classified as presenting with challenging behaviour;
- current psychotropic medication grouped into the following as per BNF criteria into ADHD/antipsychotics/antidepressants/mood stabilisers/benzodiazepines and their dosages.

### Analysis

Pearson's chi-square test was conducted for different mental disorders and challenging behaviour between the two groups at baseline. The *z*-statistic (two tailed) was used to compare the number of PwID in London and Cornwall who had medication dose changes between 2020 and 2021. Significance was accepted at *P* < 0.05.

Further, to provide insight about the degree of medication change, the total percentage change for each medication for each individual person was calculated by deducting the baseline dose (2020) from their final dose (2021) and dividing it by the total maximum permitted BNF dose. The percentage change for all individuals on the drug in each group was totalled and divided by the number of PwID on the same medication and presented as the summated percentage increase or decrease for each group. A subgroup analysis of antipsychotics and antidepressants for those with ADHD and ASD was also conducted.

## Results

There are many similarities and fewer differences in service delivery models between the two services. These are shown in Appendix 1. This therefore gives confidence in allowing for comparison between the two services. The London cohort had 113 PwID and the Cornwall cohort 97 PwID who met the inclusion criteria. Full demographic details are in Table 1. Both groups were comparable at baseline regarding level of intellectual disability, however, the male to female ratio was higher in Cornwall compared with London.

**Table 1:** Baseline demographics comparison between London and Cornwall

	London (*n* = 113)	Cornwall (*n* = 97)	*P*
Age, years: mean (median), range	39 (37), 19−84	43 (43), 20−78	−
Male:female ratio (*n*)	1.45:1 (67:46)	2.46:1 (69/28)	−
Severity of intellectual disability, *n* (%)			
Mild intellectual disability	37 (33)	34 (35)	0.7707
Moderate-severe intellectual disability	76 (67)	63 (65)	0.7707
Neurodevelopmental and psychiatric concerns present at baseline (2020), *n* (%)			
Autism spectrum disorder	74 (65)	56 (58)	0.258
Attention-deficit hyperactivity disorder	37 (33)	3 (3)	**<0.001**
Severe mental illness	37 (33)	86 (89)	**<0.001**
Challenging behaviour	44 (39)	57 (59)	**0.006**
Psychotropic medications changes (2020 to 2021), *n* (%)			
Attention-deficit hyperactivity disorder medication	29 (26)	2 (2)	**<0.001**
Antidepressants	56 (50)	43 (44)	0.44726
Antipsychotics	86 (76)	73 (75)	0.88866
Benzodiazepines	46 (41)	38 (39)	0.8181
Mood stabilisers	40 (35)	8 (8)	**<0.001**

Results in bold are significant.

ASD comorbidity in London was 65% (*n* = 74) comparable with Cornwall at 58% (*n* = 56). Differences in the numbers with comorbid ADHD between London (*n* = 37) and Cornwall (*n* = 3) were significant (*P* < 0.001). The London service had a lower proportion with a diagnosis of severe mental illness (37 *v*. 86 (33% *v*. 89%), *P* < 0.001). Challenging behaviour was more prevalent in the Cornwall cohort (44 *v*. 57 (39% *v*. 59%), *P* < 0.05).

Comparison of the number of PwID taking antipsychotics, antidepressants and benzodiazepines at baseline showed no differences between the two services but ADHD medication use was greater in London compared with Cornwall (29 *v*. 2, (26% *v*. 2%) *P* < 0.001) and so too was mood stabiliser prescribing (40 *v*. 8, (35% *v*. 8%)*P* < 0.001).

**Fig. 1:**
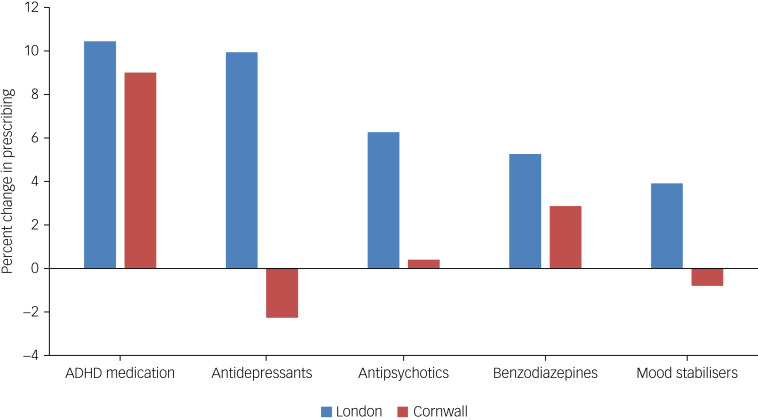
Percentage dose change in medication classes between early 2020 and early 2021 for the London and Cornwall cohorts. ADHD, attention-deficit hyperactivity disorder.

There was an overall increase in psychotropic prescribing during lockdown in urban as compared with rural settings (11% *v*. 2%). Changes in medication prescriptions over the year are displayed in Fig. 1. For London services, the largest increases were in ADHD medication (10.44%) and antidepressant dosage (9.94%). In comparison, Cornwall had a decrease in antidepressant dosage during this time (2.27%). There was also a larger increase in antipsychotic dosage in the London cohort compared with Cornwall (6.26% *v*. 0.40% increase). Overall, the Cornwall cohort remained reasonably stable with regards to medication prescribing, with no classes of medication increasing or decreasing by greater than 2.9% in total.

The further subgroup analysis for individuals with comorbid ASD taking antidepressants and antipsychotics showed no difference noted between the two centres for either medication use.

The individual drug genre changes over the year in people with common neurodevelopmental comorbidities between the two centres was compared (Table 2). For the London cohort, total average antipsychotic dose increase was 5.69% for comorbid ASD and 4.59% for comorbid ADHD. This is notably less of a change than the 9.33% for those without ASD or ADHD. Antidepressant dosage showed a 10.86% increase for those with ASD, a 1.25% decrease for those with ADHD and a 9.35% increase in those without ASD or ADHD.

**Table 2:** Comparison of percentage change in prescribing in specific subcohorts

		London, % increase or decrease		Cornwall, % increase or decrease
Antipsychotics in patients with ASD		5.69% increase		0.16% decrease
Antipsychotics in patients with ADHD		4.59% increase		12.50% increase
Antipsychotics in patients without ASD/ADHD		9.33% increase		1.34% increase
Antidepressant in patients with ASD		10.86% increase		0.48% decrease
Antidepressant in patients with ADHD		1.25% decrease		0.00%
Antidepressant in patients without ASD/ADHD		9.35% increase		4.17% decrease

ASD, autism spectrum disorder; ADHD, attention-deficit hyperactivity disorder.

In comparison, across the 1 year, in Cornwall antipsychotic prescribing for those with ASD decreased by 0.16% and increased by 1.34% for those without ASD and ADHD. Antidepressants also remained stable with a 0.48% decrease for those with ASD and 4.17% decrease for those without ASD or ADHD. As there were only two PwID with comorbid ADHD taking antipsychotics, and one taking antidepressants, results are incomparable with London because of the low sample size, however the figure has been included in Table 2 for completion.

## Discussion

This is the first study looking at psychotropic prescribing changes during COVID for PwID by comparing two centres that were affected by COVID-19 differently. A previous regional study in the UK looking at retrospective and prospective prescribing before and during lockdown concluded that COVID-19-related lockdown resulted in an increase in medication interventions, total consultations and increased involvement of multidisciplinary teams.^22^ It is an important area to explore to understand and optimise medication usage as changes in prescribing can be the result of multiple factors such as worsening of mental health during the pandemic, over-reliance on medications, the natural trajectory of the mental disorders or lack of/ less effective non-pharmacological interventions.

These two centres in two different regions were different in their geography (rural versus urban). There was no noted difference in the number of PwID who had their antipsychotic, antidepressant and benzodiazepine medication changed across the year. However, there was a noted increase in the total medication dosage over the pandemic period for the London cohort in comparison with the Cornwall cohort, in all psychotropic medication classes. This prescribing difference raises various hypotheses about the impact of COVID-19 on mental health and behavioural difficulties in PwID as well as many different factors that can be associated with it.

### Rural versus urban settings

The environmental impact on mental health and challenging behaviour in PwID is well established. PwID can find it hard to cope in more urban areas because of a reduction in space, a more compact living environment, paradoxically increased social isolation and a faster-paced routine for those around them. These factors were further exacerbated by COVID-19 because of isolation measures and the clear ‘stay at home’ messaging. As Cornwall had generally less strict local restrictions than London during 2020, patients and their carers may not have had a major disruption to their usual activities that many PwID require as part of their usual routine to remain stable. The fact that London's largest increases were in ADHD medication (10.44%) and antidepressants (9.94%) may suggest a worsening of ADHD symptoms, anxiety, low mood and agitation encountered with stronger restrictions. Ability to access open spaces in rural settings is easier during restrictions compared to urban settings, hence non-pharmacological interventions could have been a solution to increasing ADHD symptoms or worsening low mood or anxiety in rural settings.

### Service-related issues

The setup of psychiatric services and their respective mechanisms of response pre-pandemic could have played a role. In London, there is an easy and direct access to psychiatrists generally. During the pandemic, as multidisciplinary face-to-face interventions were very rarely carried out psychiatric direct involvment due to easy accessibility could have been greater. In Cornwall pre-pandemic service delivery required referrals for mental health problems to be mainly managed by the multidisciplinary team. Psychiatrists are only involved for consultations or if an acute mental health need is recognised. This is because a study in 2017 in Cornwall showcased that direct involvement of psychiatrists as opposed to engaging via the multidisciplinary team led to more likelihood of psychotropic prescribing.^23^ This led to changes in service function and psychotropic prescribing in Cornwall for PwID pre-pandemic.^24^ This model continued during the pandemic. This may have encouraged use of non-pharmacological interventions prior to pharmacological optimisation during the pandemic. Differences in mental illnesses in the participants of the study between the two services could be because of many other different factors. This could also be simply owing to varying prevalence rates of mental disorders in different parts of the country as studies have shown that prevalence rates of mental disorders can be higher in London compared with other areas.^10^

### PwID and other neurodevelopmental disorders

Given the significantly high comorbidity of mental disorders such as anxiety and mood disorders in people with neurodevelopmental disorders, mainly ASD and ADHD, a subanalysis was conducted to explore any differences in prescribing in PwID and other neurodevelopmental disorders. Authors had hypothesised that PwID who also have ASD and/or ADHD are likely to be affected more because of changes to their routine and various disruptions. However, patients with ASD and ADHD had a lower percentage increase in antipsychotics dosage in comparison to those without ASD or ADHD, in both cohorts. The picture presented with anti-depressants was more complex and diffcult to rationalise. On the whole it could be the lockdown had the reciprocal effect of narrowing social expectations and the range of activities, thus reducing anxiety and stress.

### Strengths and limitations

This is one of the earliest studies looking at psychotropic prescribing in PwID during the pandemic. Retrospective collection of data as part of a quality improvement project helped to get a snapshot of real-life changes in clinical practice. In addition to shedding light on changes to prescribing during the panemic for PwID, it also raised issues related to complex factors associated generally with psychotropic prescribing. Use of data from two different sites is a strength of this study as use of one set of data would not be representative of this complex patient group. Although similar studies can be done using large data-sets across England they lack the coalface clinical granularity such a study as this one provides. This retrospective cohort study is not without its limitations.

First, two specific specialist services were analysed and thus the data may not be generalisable to the whole of England because of prescribing patterns and service logistics that are specific to these centres. However, both services, as shown in Appendix 1, are broadly commissioned to meet the care needs of similar populations. Furthermore, each area may have differing demographics such as patient ethnicity, age, socioeconomic status, living arrangements, extent of social support, funding and physical and mental comorbidities; these were not accounted for within this study. Possible confounders include worsening mental illness that was not triggered by the effects of the pandemic. The change noticed of increase in the use of psychotropic medication in one centre could be part of an ongoing change and not necessarily the impact of COVID. This can only be only ruled out if there were data from another non-COVID period for comparison. Further limitations include the study being retrospective, practice standards and collecting data from clinic letters allowing for variation in clinical practice and clinician attributes. Although national standards (ICD/DSM) have been used with diagnoses, there may be inter-reliability issues owing to the complex nature of the patients in each cohort. There were only two PwID on ADHD medication in the Cornwall cohort, which showed an increase of 9.00%; however, this is non-generalisable and incomparable because of the sample size. This is because in Cornwall unless there is another associated comorbidity, PwID with ADHD are diagnosed and discharged back with medication advice to primary care who refer to the specialist service based on National Institute for Health and Care Excellence guidance yearly for a one-off review.

Although a clear pattern for increased prescribing was seen in London during 2020, further research is required in the year 2021 to see if there have been any changes or reduction in prescriptions following the easing of national and local restrictions.

### Implications for clinical practice

This is one of the first studies to tangibly highlight that the pandemic could have caused an increase in psychotropic prescribing for PwID in some parts of the country. Studies have shown a similar increase in psychotropic prescribing in older people with dementia.^25^ This highlights the need for ongoing specialist multidisciplinary input, particularly following the effects of the pandemic as there can be an over-reliance on medications to compensate for gaps in alternative interventions. Therefore, it is important for the whole system to work together for any psychotropic medication used during the pandemic or considered in future to be optimised.

### Implications for research

This study looks to ascertain if there are differences in psychotropic prescribing in an area impacted significantly by COVID versus an area where there was a minimum impact of COVID. There are trends to suggest higher levels of psychotropics could have been used in areas where the pandemic has affected more. This study could be the pre-cursor for a data-linked study to identify prescribing changes in COVID high impact versus COVID low impact areas. This is important as the last 6 years in England there has been a focus on medication optimisation and deprescribing for PwID. The pandemic could be a negative catalyst in reversing this in COVID high impact zones.

### Implications for policy

This study again highlights the complex issue of prescribing psychotropic medications for PwID. A multitude of factors influence prescribing of psychotropic medications, which are not easy to unpick at the best of times. Recent studies^24,26^ and the recent Royal College of Psychiatrists report^27^ highlight the complexity of the problem of high usage of psychotropic prescribing in PwID. However, these have not taken into consideration the added impact of the pandemic. Any future policies and recommendations looking at rationalising psychotropic prescribing in PwID need to take into account the pandemic as there will be implications on needed resources and training over and above the usual influencing factors for prescribing psychotropics in this vulnerable population. In particular, considerations need to be given to the additional vulnerabilities that PwID living in highly urban environments face, as suggested by our findings.

## Data Availability

Data releavent for this study can be obtained from the corresponding author if the request is reasonable.

## Supplementary material

For supplementary material accompanying this paper visit http://dx.doi.org/10.1192/bjo.2021.1064. click here to view supplementary material

## Appendix

**Table:** Comparison of psychiatric services for adults with intellectual disabilities in London and Cornwall

London	Cornwall
Two consultant psychiatrists with a total of 15 clinical sessions/week focused on managing mental health and challenging behaviour concerns	Three consultant psychiatrists with a total of 15 clinical sessions/week focused on managing mental health and challenging behaviour concerns
Three trainee doctors, MDT team with psychologists, LD nurses, occupational therapists, speech and language therapists, social workers, physiotherapists	Psychiatrists support 2 × MDT teams with psychologists, LD nurses, liaison nurses, epilepsy nurses, occupational therapists, speech and language therapists, dietitian, liaison social worker
Integrated service where health and social care work together	Social care and LD services not integrated
Easy access to psychiatrists through direct referrals routes	Work to a ‘pathways' model where the psychiatrist provides consultation based on presenting mental health need based on prior screening done by care co-ordinator using tools such as Moss – Psychiatric Assessment Schedule (intellectual disability) and Glasgow Depression Scale

LD, learning disability; MDT, multidisciplinary team.
